# The HIV Tat protein affects processing of ribosomal RNA precursor

**DOI:** 10.1186/1471-2121-9-32

**Published:** 2008-06-17

**Authors:** Donatella Ponti, Maria Troiano, Gian Carlo Bellenchi, Piero A Battaglia, Franca Gigliani

**Affiliations:** 1 Dipartimento di Biotecnologie Cellulari ed Ematologia, Università La Sapienza, Roma, Italia; 2 Dipartimento di Biologia Cellulare e Neuroscienze, Istituto Superiore di Sanità, Roma, Italia; 3 Istituto di Genetica e Biofisica "Adriano Buzzati Traverso", CNR, Napoli, Italia

## Abstract

**Background:**

Inside the cell, the HIV Tat protein is mainly found in the nucleus and nucleolus. The nucleolus, the site of ribosome biogenesis, is a highly organized, non-membrane-bound sub-compartment where proteins with a high affinity for nucleolar components are found. While it is well known that Tat accumulates in the nucleolus via a specific nucleolar targeting sequence, its function in this compartment it still unknown.

**Results:**

To clarify the significance of the Tat nucleolar localization, we induced the expression of the protein during oogenesis in *Drosophila melanogaster *strain transgenic for HIV-*tat *gene. Here we show that Tat localizes in the nucleoli of *Drosophila *oocyte nurse cells, where it specifically co-localizes with fibrillarin. Tat expression is accompanied by a significant decrease of cytoplasmic ribosomes, which is apparently related to an impairment of ribosomal rRNA precursor processing. Such an event is accounted for by the interaction of Tat with fibrillarin and U3 snoRNA, which are both required for pre-rRNA maturation.

**Conclusion:**

Our data contribute to understanding the function of Tat in the nucleolus, where ribosomal RNA synthesis and cell cycle control take place. The impairment of nucleolar pre-rRNA maturation through the interaction of Tat with fibrillarin-U3snoRNA complex suggests a process by which the virus modulates host response, thus contributing to apoptosis and protein shut-off in HIV-uninfected cells.

## Background

The HIV-1 Tat protein is an RNA binding protein essential for viral replication [1], which regulates productive and processive transcription from the HIV-1 long terminal repeat (LTR) by binding to the transactivation response (TAR) element [2]. Tat also affects several cellular functions by inducing angiogenesis [3,4], cell proliferation and apoptosis [5]. Moreover, Tat regulates cytokine gene expression [5,6], immune cell activation and may be secreted by HIV-1 infected cells, and thus act on neighboring cells [7-9]. Furthermore, by interacting with tubulin [10,11], Tat affects mitosis and induces aneuploidies [12].

It has been shown that in both infected and uninfected cells, Tat also localizes in the nucleolus [13] and that the nucleolar accumulation of Tat occurs via the highly conserved stretch of basic amino acids that acts as a nucleolar localization signal (NoLS) [14-17].

The nucleolus is a highly structured and dynamic organelle involved in the transcription and maturation of rRNA and ribosome biogenesis as well as in apoptosis and in cell cycle control [18]. Recently, various studies have suggested that the nucleolus plays a role in HIV-1 infections. First, lymphocytes isolated from infected patients show abnormal nucleolar structures [19]. Secondly, nucleolar localization of TAR impairs virus progression [20,21].

A number of proteins encoded by RNA viruses, such as protein V of adenovirus [22], protein N of porcine virus [23] and HIV-1-Rev protein [24], are known to enter the nucleolus and to colocalize with nucleolar components suggesting that nucleolar localization is an important step in promoting viral replication [25]. While it is known that Rev multimerizes in the nucleolus and appears to play a role in the nucleoplasmic transport of viral unspliced RNAs [26]; the function of Tat in this nuclear structure is still unknown.

To gain insight into the functional significance of Tat nucleolar localization, we used a *Drosophila melanogaster *Tat transgenic strain [10,12] and analysed the effect of this protein on ribosome biogenesis by expressing it (under the control of hsp70 promoter) during *Drosophila *oogenesis. *Drosophila *oogenesis is, in fact, the period of highest ribosome biosynthesis, and the genetic and molecular events (highly conserved from yeast to human) of this process have been extensively studied and described in detail [27-29]. Furthermore, recent data indicate that *Drosophila *is a useful complementary system for studying HIV-1-related genes and proteins [10,30,31].

Here we show that Tat localizes with fibrillarin in nurse cell nucleoli, causes death and delayed development of the progeny and decreases the level of the 80S ribosome particles. This decrease appears to result from a Tat induced inhibition of pre-rRNA processing caused by the interaction of Tat with U3 snoRNA and fibrillarin, nucleolar components that are essential for the early steps of pre-rRNA processing. The results reported in this paper, provide the first evidence of the action of Tat on nucleolar functions and further our understanding of the molecular mechanism underlying Tat-mediated pathogenesis.

## Results

### Tat expressed by heat shock treatment, colocalizes with fibrillarin in the nucleolus of Drosophila cells

**Figure 1:**
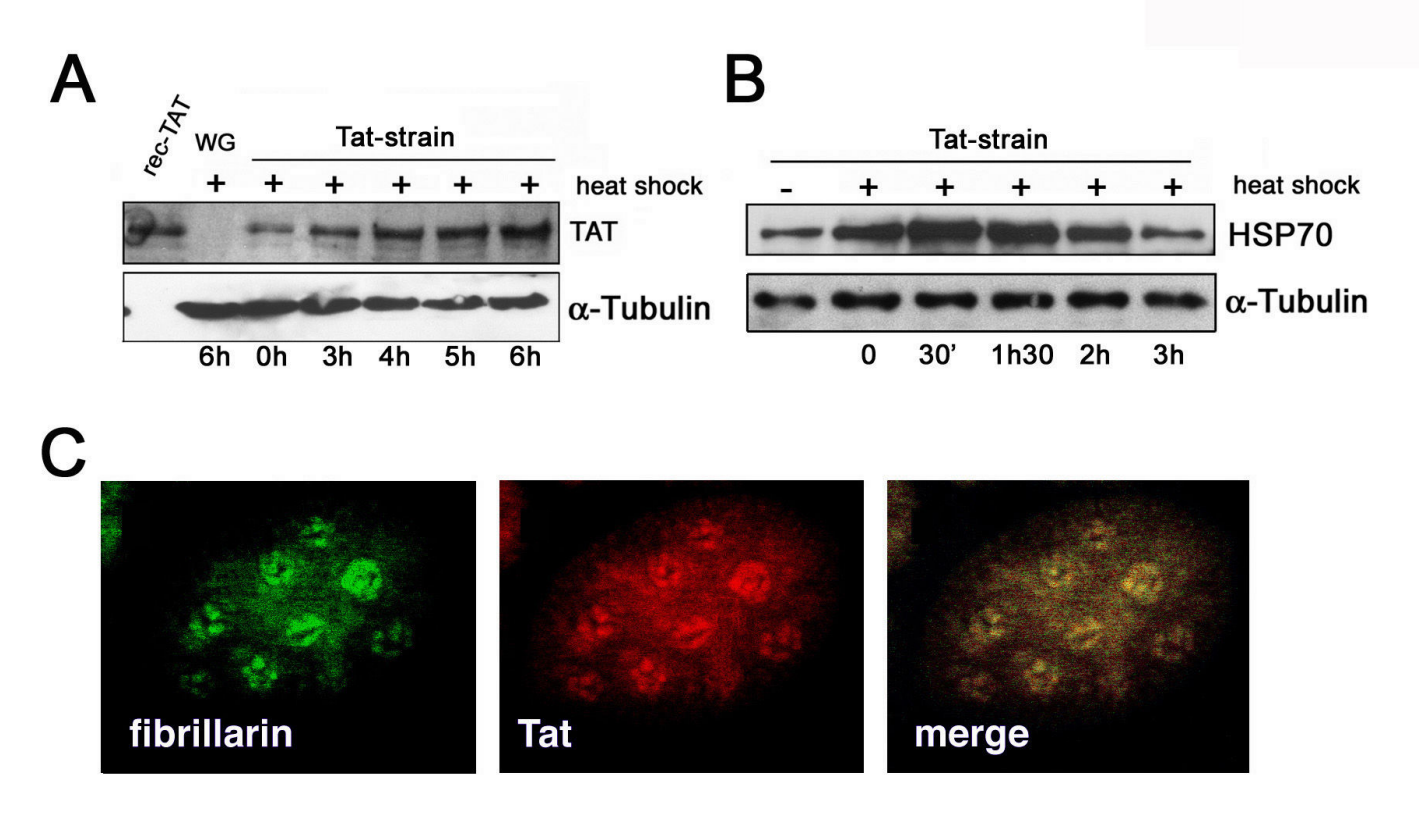
**Expression of Tat and its colocalization with fibrillarin in *Drosophila *nurse cell nucleoli from Tat transgenic strain after heat shock treatment**. Protein extract from Tat transgenic strain at different time-points, up to six hours after heat shock were analysed by western blot (A). Control (WG six hours after treatment) and recombinant Tat are shown. Expression of HSP70 protein in Tat transgenic flies, at different times after heat-shock treatment (+) is shown in B, as well as levels of HSP70, in flies not subjected to heat-shock (-). α-tubulin is shown as the control in both panels. Confocal images (C) from *Drosophila *nurse cells double labelled with anti-fibrillarin (green), and anti-Tat (red); merged image reveals the colocalization of Tat with fibrillarin in the nurse cell nucleoli after heat shock treatment.

Nucleolar localization of the HIV Tat protein has been extensively shown using several cellular systems [13-17]. To shed light on the effects of the nucleolar localization of Tat, we determined whether it localizes in the nucleolus of *tat *transgenic *Drosophila melanogaster *cells, where its expressions is driven under the control of the heat shock promoter [10]. Since it is known that heat shock affects ribosome biogenesis and its effects terminate four hours later [32], we first investigated the time conditions that allow high expression of Tat in the absence of heat shock treatment effects (see Materials and Methods). To this end, we analyzed the expression of both Hsp70 and Tat proteins by Western blot analysis. Results in Fig. 1A show that the levels of Tat protein rise soon after heat shock treatment and continue for six hours during the ensuing treatment. In contrast, an increased level of Hsp70 protein appears between 30 minutes and two hours after treatment (Fig. 1B). Based on these results, all analyses were carried out by subjecting *Drosophila *flies to three heat shocks and by starting experiments six hours thereafter. Further to focusing on Tat nucleolar localization, *Drosophila *nurse cells were used since they are relatively large (due to polyploidy) and each contains several nucleoli. The images in Fig 1C show that Tat expressed in *Drosophila *transgenic flies localizes, as expected, both in the nucleoplasm and in the nucleolus, where it colocalizes with the nucleolar marker fibrillarin. This result supports the idea that the nucleolar localization signal works in *Drosophila *as well as mammalian cells.

Since the nucleolus is the site of ribosome RNA synthesis, we investigated whether Tat expression gives rise, in *Drosophila*, to phenotypes affecting ribosome biogenesis such as death and delayed development [28]. Therefore, to test whether the expression of Tat gives rise to similar phenotypes, we expressed this protein during *Drosophila *oogenesis and checked the progeny for mortality and developmental time. The data show that the expression of Tat causes significant increased mortality at embryonic and larval stages (Table 1). Moreover, developmental stages were delayed (4 days) compared with the control strain. These results indicate that Tat may affect ribosome biogenesis.

**Table 1:** Number of embryos, larvae and adults from *Drosophila *WG (control) and Tat strains after heat shock treatment. Results are means ± S.D. (*n *= 6); *P*< 0,001

	**Viability of Tat strain**		
**Strain**	**Embryos**	**Larvae**	**Adults**

**WG**	216,7 ± 13,6	63,5 ± 5,3	59,5 ± 4,2
**Tat**	186,7 ± 17,9	43,6 ± 6,2	40,6 ± 5,7

### Tat reduces the level of 80S ribosomes

**Figure 2:**
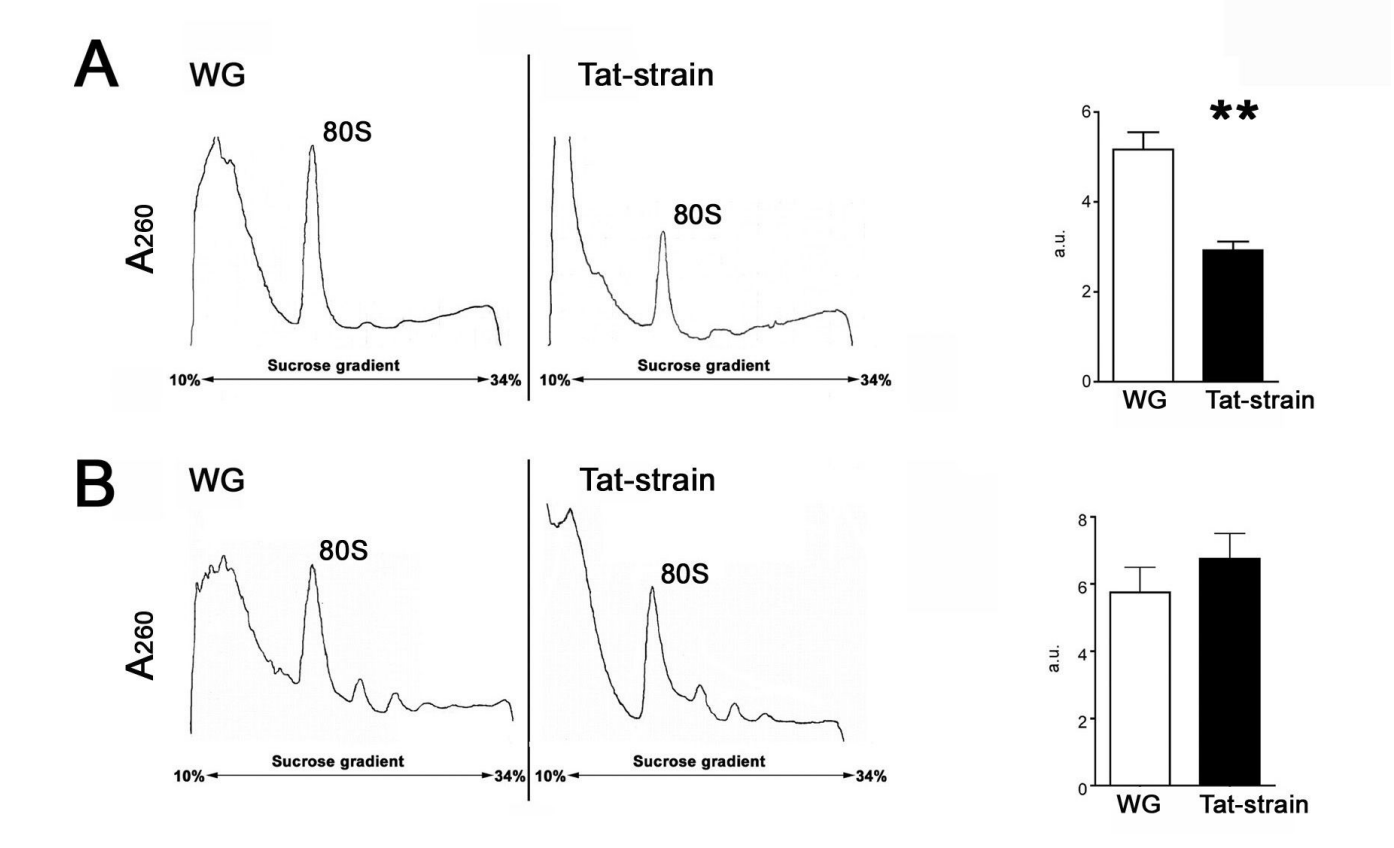
**Tat affects ribosomal profiles from *Drosophila *female extracts**. Ribosomal profiles of control (WG) and transgenic (Tat-strain) strains, subjected (A) and not (B) to heat shock treatment (equal amount of O.D.260 were loaded). Histograms show quantification of 80S particle, (** p < 0.01).

Based on the above results, we tested whether Tat interferes with ribosome biogenesis by analysing the ribosome distribution profiles of both transgenic and control flies subjected to three heat shock treatments. Sucrose gradient profiles in Figure 2 show that, under these conditions, post mitochondrial supernatant (PMS) from Tat-transgenic flies (Fig. 2A right) contains a significantly lower amount of 80S ribosomes than comparable samples from control flies (Fig. 2A left). As expected, no significant difference was observed in strains not subjected to heat shock (Fig. 2B). Histograms represent the average area, obtained from 3 independent experiments, of the 80S ribosome peak. Significant differences (p < 0.01) were detected only in Tat-transgenic flies after heat shock treatment.

### Tat interferes with pre-rRNA processing patways

**Figure 3:**
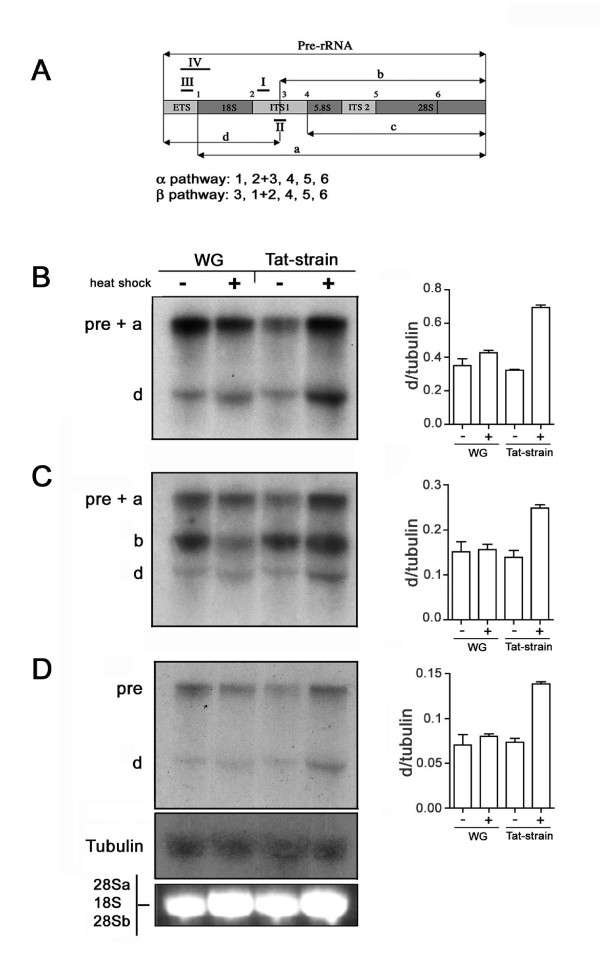
**Tat affects rRNA processing**. (A): scheme of *Drosophila *rRNA precursor with cleavage sites corresponding to the two alternative processing pathways, α and β. Sequences of cleavages generating the two different pathways are highlighted under the scheme (see reference 29 for detail). Solid bars show probe positions. (B-D): Northern blot of RNA extract from control (WG) and transgenic strains (Tat-strain) with (+) or without (-) heat shock treatment. Probe I, derived from the ITS1 region and closed to cleavage site 2, was used in B. The same blot was hybridized using respectively probe II (C) derived from the ITS1 region including cleavage site 3 and probe III (D) derived from the ETS region, closed to cleavage site 1. The different immature products are indicated on the left (see scheme 4A). Tubulin is shown as loading control. Histograms represent the average from three independent experiments.

In order to assess whether Tat interferes with ribosome biogenesis at the nucleolar level, we investigated the effect(s) of Tat on pre-rRNA processing. In *Drosophila*, processing of the rRNA primary transcript (pre-rRNA) proceeds by two alternative (α and β) cleavage pathways [27,29]. In the α processing pathway (shared by all Eucarya) the initial cleavage step removes the ETS, leaving a large molecule encompassing both the 18S and 28S rRNA sequences (intermediate a) (Fig. 3A). In the alternative β pathway, the primary transcript is cleaved inside the ITS region, at site 3, generating two processing intermediates; the first containing the 18S rRNA precursor (intermediate d), and the second containing the 28S rRNA precursor (intermediate b) (Fig. 3A). The effect of Tat on pre-rRNA processing was investigated by Northern blot analysis using total RNA extracted from *Drosophila *females of both transgenic and control strains subjected and not to heat shock treatment. The presence of unprocessed rRNA precursors in Tat-expressing cells was investigated using a DNA probe (probe I) which specifically hybridizes with the ITS1 5' region (Fig. 3A), thus allowing the identification of unprocessed pre-rRNA and both the a and d precursors. As Fig. 3B shows, hybridization of total RNA from Tat-expressing and control flies with the ITS1-specific probe clearly reveals accumulation of type d precursor only in Tat-expressing flies. Therefore, Tat expression in *Drosophila *seems to affect at least cleavage site 1. With the α pathway blocked, pre-rRNA processing should proceed mainly through pathway β, generating increasing amounts of intermediate d [29]. Indeed, hybridization with probe II shows higher levels of both b and d intermediate forms in the Tat expressing strain (Fig. 3C), suggesting that both pathways may be affected. The observation that Tat mainly interferes with site 1 cleavage was further confirmed by Northern blot experiments using a DNA probe (probe III) that specifically hybridizes with the 5' ETS region (Fig. 3A), thus allowing detection of accumulated intermediate d forms. As Fig. 3D shows, a significant increase of d form RNA occurs only in transgenic flies expressing Tat, which confirms that Tat affects at least cleavage site 1 impairing pre-rRNA maturation.

### Tat binds to ETS-18S region of pre-rRNA

**Figure 4:**
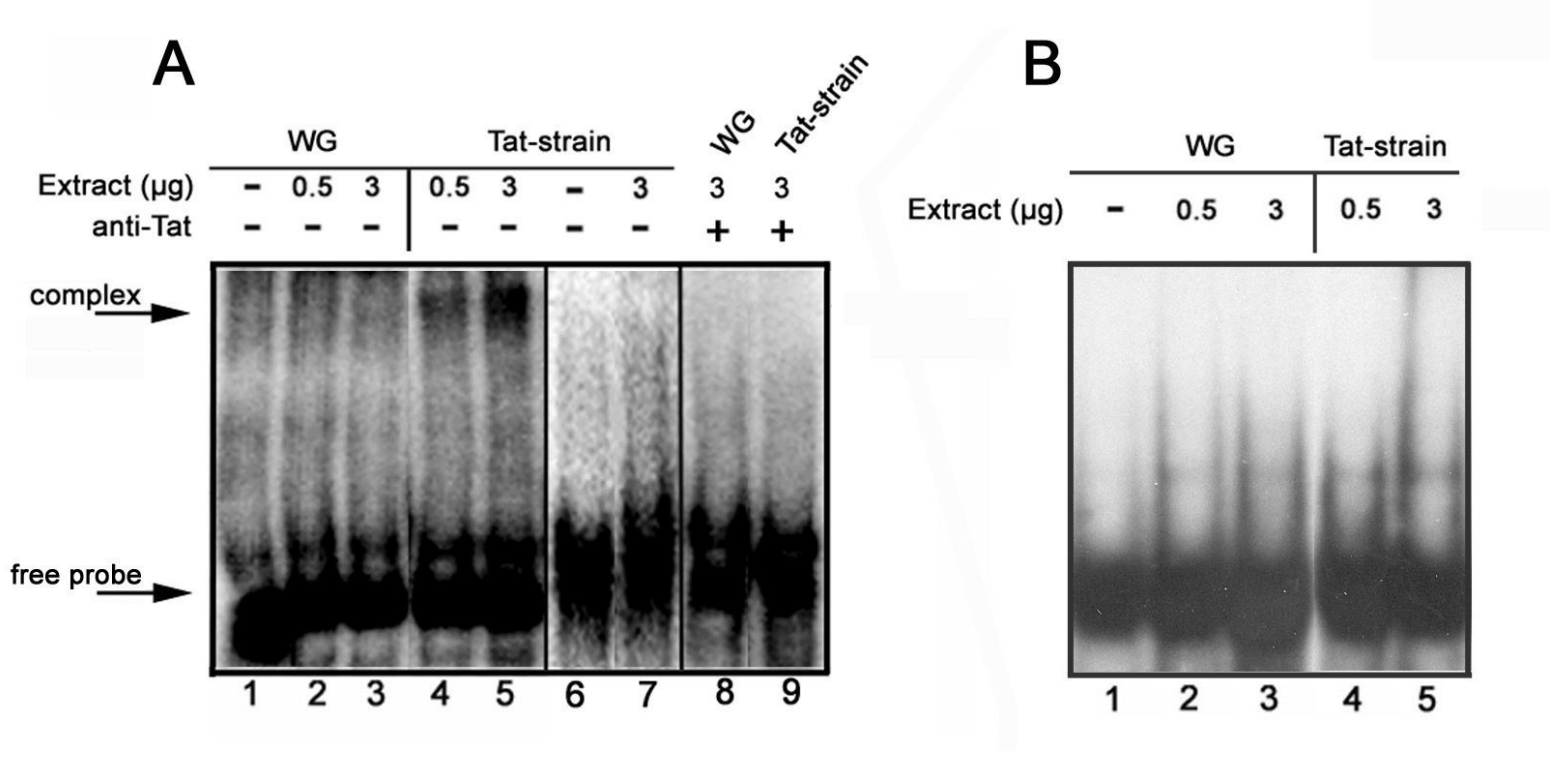
**Tat binds to the ETS-18S region**. Electro mobility shift assay (EMSA) using as the probe the region corresponding to the ETS-18S pre-rRNA, containing cleavage site 1 (probe IV in Fig. 3A). (A): nuclear extracts from control (WG), and transgenic (Tat-strain) strains were incubated, after heat shock treatment, with the ETS-18S probe. Lane 1, free probe. In lanes 2 and 3, the probe was incubated with 0.5 and 3 μg of nuclear extract from control strain. In lanes 4 and 5 the probe was incubated with 0.5 μg and 3 μg of nuclear extract from transgenic strain. Unspecific probe, was incubated (lane 7) or not (lane 6) with nuclear extract from transgenic strain. In lane 8 and 9 nuclear extracts were pre-incubated with the anti-Tat antibody and then with the probe. (B): Nuclear extracts from control (WG), lanes 2–3, and transgenic (Tat-strain) strains, lanes 4–5, were incubated, after heat shock treatment, with an unrelated structured probe (tRNA). Probe alone is shown in lane 1.

To understand whether Tat protein interacts with pre-rRNA, we performed EMSA using an RNA stretch comprising cleavage site 1 of ETS-18S rRNA (probe IV in Fig. 3A) as the probe. We incubated nuclear extracts from control and transgenic strains with probe IV after heat shock treatment. In contrast to samples from non-Tat expressing flies (Fig. 4A, lanes 2 and 3), incubation with extracts from Tat expressing flies resulted in considerable retardation of probe electrophoretic mobility (Fig. 4A, lanes 4 and 5). The shift was abolished after incubation the extracts with anti-Tat antibody (lanes 8 and 9) before adding the probe. To further confirm the specificity of the interaction extracts were incubated with a random RNA probe transcribed from cloning vector sequence (Fig. 4A, lanes 6 and 7) and with an unspecific structured probe (tRNA) (Fig. 4B). No shift was observed in any of these cases.

### Tat in vivo interacts with components involved in the first step of pre-rRNA processing

Fibrillarin is one of the essential components involved in pre-rRNA maturation. It localizes throughout the nucleolus and in the fibrillar region of mammalian nucleoli [33]. Since Tat colocalizes with fibrillarin in *Drosophila *transgenic strains (Fig. 1C), we next investigated whether Tat interferes with pre-rRNA processing by interacting with fibrillarin.

**Figure 5:**
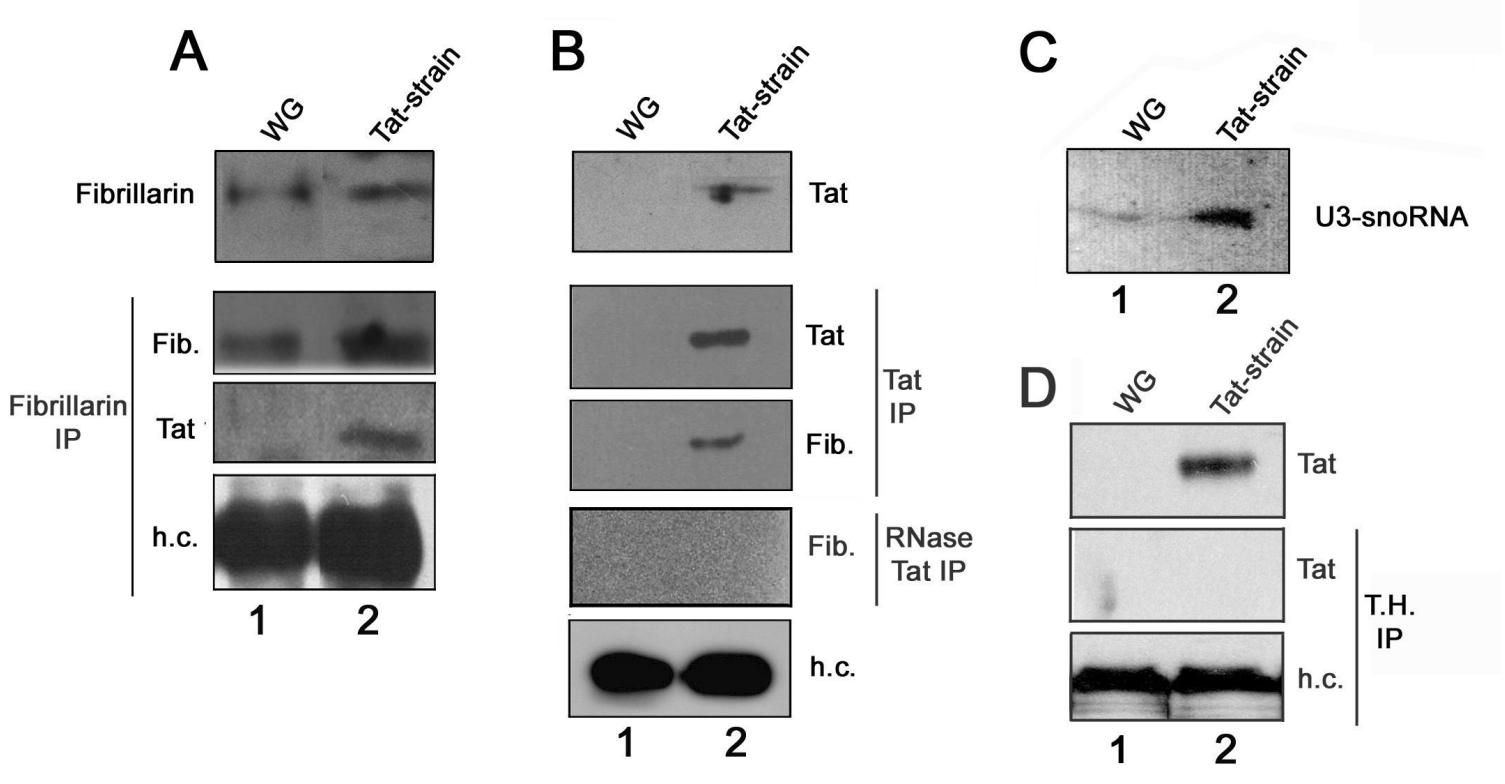
**Tat interaction with the fibrillarin-U3 snoRNA complex**. Extracts from control flies (WG) (lane 1), and Tat-transgenic flies (Tat-strain) (lane2), following heat-shock treatment. (A) From top to bottom: nuclear lysate probed with anti-fibrillarin antibody; anti-fibrillarin immunoprecipitate probed with anti-fibrillarin antibody; anti-fibrillarin immunoprecipitate probed with anti-Tat; antibody; heavy chain (h.c.) are shown as loading control. (B) From top to bottom: Nuclear lysate probed with anti-Tat; anti-Tat immunoprecipitate probed with anti-Tat antibody; anti-Tat immunoprecipitate probed with anti-fibrillarin antibody; nuclear lysate immunoprecipitated with anti Tat antibody, after RNase treatment, and than probed with anti fibrillarin antibody; antibody heavy chain (h.c.) are shown as loading control. (C) Northern blotting of nuclear extract immunoprecipitated with anti-Tat and probed with U3 specific oligonucleotide. (D) Immunoprecipitation with an unrelated antibody. From top to bottom: nuclear lysate probed with anti Tat antibody; nuclear lysate immunoprecipitated with anti tyrosine hydroxylase (T.H.) and probed with anti Tat antibody; antibody heavy chain (h.c.) are shown as loading control.

To this end, nuclear protein extracts were immunoprecipitated with anti-fibrillarin polyclonal antibodies and challenged with anti-Tat polyclonal antibodies (Fig. 5A). The specific signal was detected in the Tat-expressing strain (lane 2), whereas, as expected, no signal was observed in the control strain (lane 1). Similarly, when nuclear extracts were immunoprecipitated with anti-Tat antibodies and then challenged with anti-fibrillarin antibodies (Fig. 5B), the immunoreactive band was detected only in precipitates from the Tat-expressing strain (lane 2). Interestingly the detection was abolished when samples were preincubated with RNase before proceeding with the immunoprecipitation (Fig. 5B, bottom lanes). Since fibrillarin is known to participate in RNA cleavage in association with U3 snoRNA, we tested whether Tat forms immunoprecipitates with U3 snoRNA [34,35]. Accordingly, we incubated nuclear extracts with Tat-polyclonal antibodies and then monitored the presence of a Tat-U3 complex by Northern blotting using a U3 specific oligonucleotide as a probe. Results in Fig. 5C (lanes 1 and 2), clearly show that U3 is associated with Tat in immunoprecipitates from a transgenic strain expressing Tat. Specificity of the immunoprecipitation was confirmed performing the experiment with an unrelated antibody (anti Tyrosine hydroxylase). In such case no specific Tat signal was detected (Fig. 5D). On the whole, the results in Fig. 5 suggest that the association of Tat with fibrillarin and U3 snoRNA impairs pre-rRNA processing.

## Discussion

In this study, we show that Tat expressed in *Drosophila *colocalizes with fibrillarin in nurse cell nucleoli, produces a significant decrease of cytoplasmic ribosomes and affects cleavage sites during RNA precursor maturation.

In Jurkat T cells, it has been shown that Tat localizes throughout the nucleolus and, by electron microscopy, has been visualized in the dense fibrillar (DFC) and granular (GC) nucleolar components [33] where pre-rRNA processing mainly occurs. Therefore, we surmise that Tat affects ribosomal RNA maturation by interfering with post-trascriptional modifications and/or interacting with molecules involved in cleavage events. The result in Fig. 5 indicates that Tat immunoprecipitates with fibrillarin and with small nucleolar U3 RNA. The interaction of Tat with fibrillarin seems to be mediated by the presence of RNA based components cause it is abolished after RNase treatment. However we cannot exclude that RNase treatment compromise the integrity of the complex, and than impair protein-protein interaction.

Fibrillarin (highly conserved among eukaryotes) is a component of C/D box small nucleolar ribonucleoproteins (snoRNPs) and directs 2'-O-methylation of rRNA. In association with U3, U8, and U13 small nuclear RNAs, it is involved in the first step of pre-rRNA processing [34,35]. The processing and modification of ribosomal pre-RNA in the nucleoli of eukaryotic cells, is accomplished by a large number of small nucleolar RNAs (snoRNAs) complexed with proteins in ribonucleoproteic particles (snoRNPs) that are essential for rRNA chemical modifications, such as methylation and pseudouridylation [36,37]. Defects in some of these components have been associated with defects in pre-rRNA processing [29,38]. Thus, Tat can inhibit or delay the processing of the pre-rRNA by subtracting fibrillarin-snoU3 complex from the initial cleavage site. Alternatively Tat could interact: i) with specific RNAs and drag along fibrillarin and other core components of the snoRNP (such as Nop56, Nop58, 15.5 K); ii) with proteins that interact with snoRNPs (such as Nopp140 or nucleolin); iii) or directly with the rRNA. This should affect fibrillarin functions in cells harbouring Tat. Interestingly, a similar mechanism has been suggested for Nidoviruses, which are able to traslocate the nucleocapside (N) protein into the nucleolus, where it interacts with fibrillarin, by an RNA mediated mechanism and possibly affects pre-RNA processing [23].

It is known that in mammalian cells, cell cycle regulation is related to ribosome biogenesis at nucleolar level [39], and that inhibition of pre-rRNA processing directs the cells toward apoptosis. Data report that mutation in nucleolar components results in pre-rRNA maturation defects and apoptosis. It is the case of mutation of *minifly *gene (which encodes an ubiquitous nucleolar protein (Mfl) in *Drosophila *[29]), and mutation in the gene encoding ribosomal RPS19 protein in humans [38]. Therefore, one of the molecular mechanisms by which both HIV infected and uninfected cells undergo apoptosis may depend on the pre-rRNA processing inhibition mediated by Tat.

Modulating cellular protein synthesis and host cell metabolism is a strategy common in many viruses to induce cellular shut-off [40]. A mechanism by which HIV-1 induces cellular shut-off seems to depend on the binding of the second coding exon of Tat to the translation elongation factor 1-delta (EF-1δ) [41]. As we observed, the inhibition of pre-rRNA processing mediated by Tat leads to a decreased amount of 80S ribosome particles. The decrease of the cytoplasmic ribosomes should result in reduced protein synthesis of the host cell. Therefore, the inhibition of pre-rRNA processing caused by Tat delineates a novel mechanism for HIV-1 mediated cellular shut-off.

## Conclusion

In the present work we show for the first time that Tat, in the nucleolus, affects ribosome rRNA maturation. This suggests that Tat manipulates basic cellular processes in order to control ribosomal biogenesis.

An intriguing hypothesis is that Tat, secreted by HIV-1 infected cells, acts on the nucleolar compartment of neighbouring uninfected cells, inducing apoptosis and consequent loss of immune competence, which is the main cause of AIDS pathogenesis.

## Methods

### Drosophila stock and transgenic line

Fly stocks: *w*^67^*c*^23 ^used as control (named WG) and WG hsp:Tat transgenic line (named Tat-strain) [10].

### Expression of HIV-Tat protein by heat shock treatment

In all experiments, Tat expression (under the control of hsp70 promoter) was induced by subjecting *Drosophila *females to heat-shock treatment for 30' at 37°C for three days. To test the viability and developmental time of strain expressing Tat, *Drosophila *virgin females (1 day old) of both transgenic and control lines were subjected to heat shock treatments and then mated. For each strain laid embryos were collected and counted at 24 and 48 hours. The time for different developmental stages (from embryo to larva and from larva to pupa) was measured. The experiment was repeated five times. Statistical analysis was performed with Graph-pad Prism software. To determine the time conditions of Tat expression, synchronized *Drosophila *females, one day old, were subjected to the heat shock procedure described above and collected at different times at 25°C. The protein extracts were analysed by Western blotting to detect Tat and Hsp70 proteins.

### Immunofluorescence microscopy

Ovaries, extracted after 6 hours following the last heat shock, in the presence of 1× modified Robb's medium (MRM) [42], were dissected in PBS buffer containing 5% glycerol, incubated with 100% methyl alcohol for 10' at room temperature and then 30' at -20°C. The same treatment was repeated with 100% acetone. After washing with PBS containing 3% BSA, the ovaries were incubated overnight at 4°C with 40% goat serum and then with rabbit anti-Tat (kindly provided by E. Loret) and/or mouse anti-fibrillarin (abcam 38F3 Cambridge, UK) both diluted 1:700 overnight at 4°C. Secondary antibodies conjugated respectively with rhodamine and FITC (Cappel) were both diluted 1:500. Samples were analysed with a Leica confocal.

### Western blotting and Immunoprecipitation

To detect the expression of Tat in Tat-transgenic *Drosophila*, 1-day old females were subjected to heat shock treatment (see section above), and protein extracts were fractionated by 15% SDS-polyacrilamide gel (PAGE) elecrophoresis and transferred to PVDF sheets (Biorad). Membranes were incubated in 10% of no fat dry milk (Biorad) in NET buffer [43] overnight at 4°C. After incubation, the sheets were washed in NET buffer and then incubated with both anti-Tat antibody at a dilution 1:2000 for 4 h and Hsp70 antibody (1: 1000 dilution; Stressgene), at room temperature. Blots were then washed and incubated with secondary antibody (goat anti-rabbit) conjugated to horseradish-peroxidase (Biorad, 1:10,000). The reaction was visualised by incubation with ECL chemiluminescence reagent (Biorad). For immunoprecipitation experiments, an equal amount of nuclear protein extracts (as further described in the EMSA assay) [44] were immunoprecipitated with anti-fibrillarin antibody (kindly provided by I. Bozzoni), with anti-Tat antibody or with anti Tyrosine hydroxylase antibody (Chemicon), using protein A/G plus-agarose (Santa Cruz) as recommended by the manufacturer. For RNase treatment extracts were incubated in presence of 40 μg/ml of RNaseA (Fermentas). Precipitated proteins were resolved by 12% SDS-PAGE and immunoblotted with anti-Tat or anti-fibrillarin antibodies (Abcam, Cambridge). Secondary antibodies used to detect Tat and fibrillarin were goat anti-rabbit (Biorad, 1:10,000) and goat anti-mouse (Biorad, 1:10,000), respectively. For Northern blot experiments immunoprecipitates were resolved by 7% acrylamide gel, containing 8 M urea and TBE buffer [43] and filters hybridised with U3 oligonucleotide (5'CGGGATCCACCACTCAGAATTCGCTCTATC CG-3'), complementary to 3' end of *Drosophila *U3 snoRNA (Dm-818/Dm-830).

### Sucrose gradient sedimentation

Ribosomal profiles were analysed using post-mitochondrial supernatant (PMS) [45]. Briefly, 50 *Drosophila *females were homogenized in 0.5 ml of buffer (50 mM Tris, pH 7.4, 80 mM KCl, 10 mM MgCl_2_, and 0.25 M sucrose) at 0°C. After 30' centrifugation at 13000 rpm, 4°C, supernatant was collected and nucleic acid concentration was measured by spectrophotometric determination (Beckman DU 530). An equal A_260 _of PMS was loaded on 10% to 34% linear sucrose gradient containing: 50 mM Tris pH 7.4, 80 mM KCl, 10 mM MgCl_2_. Samples were centrifuged at 35.000 rpm at 4°C for 1 h, 45' (Beckman LE-80 K, rotor SW41) and analysed by density gradient fractionator (Isco Model UA-5). Statistical analysis was performed with Graph-pad Prism software.

### Northern blot analysis

Total RNA was extracted from 50 *Drosophila *females after heat shock treatment. The insects were homogenized in 0.6 ml of lysis buffer (7 M urea, 0.35 M NaCl, 0.1 M Tris-Hl pH 8, 10 mM EDTA pH 8, 2% SDS). After phenol extraction RNA was precipitated first with ethanol and than with 2 M LiCl. For Northern blot analysis [43] 10 μg of total RNA were electrophoresed and transferred to Nytran Super Charge (Schleicher and Schuell) filters for hybridisation.

The following probes were used: Probe I corresponds to oligonucleotide 5'-GTTAAAATCTTTTTATGAGGTTGCCAAGCCCCACAC-3' and probe II corresponds to oligonucleotide 5'-CACCATTTTACTGGCATATATCAATTCCTTCAATAAATG-3' (Fig. 4A). Both were phosphorilated with [γ-P^32^] at 5' terminal using T4 Kinase (Roche). Probe III (to identify 5'ETS) was amplified by PCR from genomic DNA of *Drosophila melanogaster *(using as primers ETS2up 5'CCCGGATCCGTCAAGTTTCTATTATACATAGA3' and ETS2dw 5'-GCTCTAGATATAACATAAAACCGAGCGCACATGATAATTCTT-3') and phosphorilated with [α-P^32^] by random priming (Boehringer). Probe for tubulin was generated with random priming kit (Roche). Three independent experiments were performed for each probe. Bands were quantified with a phosphoimager (Packard) and graphs plotted with Graph-pad Prism software.

### Electro mobility shift assay (EMSA)

800 nucleotides of the ETS-18S region (probe IV) were obtained by PCR from genomic *Drosophila *DNA using the following primers: 5'-ETS2up 5'-ACGGATCCGGTAGGCAGTGGTTG-3' and 18Sdw 5'-ACGCGTCGACTAATGATCCTTCCGGAGG-3'. The sequence cloned into pBSK was transcripted with T7 polymerase and labelled with [α-UTP^32^]. Unspecific probe, (900 nucleotides) was obtained from linearized pBSK, following transcription. tRNA labelled with [γ-ATP^32^] was used as the unspecific structured probe.

Nuclear extracts from 30 females, subjected and not to heat shock treatment, were obtained using the following procedure: flies were homogenized at 0°C in 0.5 ml of buffer A (1 mM Sucrose, 0.5 mM MgCl_2_, 20 mM NaCl and 20 mM Tris pH 7.4) containing 2 mM DTT, 1 mM PMSF, 50 μl of the protease inhibitor cocktail 7× (Amersham) and 20 μl of RNase inhibitor (5U/μl) (Sigma). Total lysate was first centrifuged 5' at 800 rpm 4°C to remove cellular debris and then 5' at 6000 rpm 4°C. Pellet, containing crude nuclear fraction, was homogenized in 0.5 ml PBS containing 0.2 M NaCl and 0.05% nonided-P40 and incubated 10' at 0°C. Nuclear extracts were then separated from insoluble fractions after centrifugation at 13.000 rpm 4°C. Nuclear extracts from *Drosophila *females were incubated in binding buffer (50 mM Tris-HCl pH 7.9, 200 mM NaCl, 8% glycerol), poly dI-dC (0.1 μg/μl), RNase inhibitor (5U/μl), at 0° for 10'. Then, samples were incubated with or without anti-Tat antibody at 0° C for 15' and then with 5 fmol of labelled probe (specific activity: 1.5 × 10^9^cpm/μg) at 0°C for 10'. Preparations were resolved by 4% polyacrylamide gel in non denaturating conditions, transferred onto 3 MM paper, and exposed overnight to phosphoimager (Packard).

## Abbreviations

HIV-1: human immunodeficiency virus, type 1; Tat: transcriptional activator; TAR: transactivation response; LTR: long terminal repeat; HSP: heat shock protein.

## Authors' contributions

DP conceived and performed most of the studies, MT performed northern blot analysis, GCB performed confocal analysis, FG and PAB were involved in sponsoring the project and manuscript preparation. All the authors have read and approved the final manuscript.
